# Validity and Reliability of the Malay Questionnaire for Olfactory Disorders

**DOI:** 10.21315/mjms2023.30.6.15

**Published:** 2023-12-19

**Authors:** Erica Gima, Carren Sui Lin Teh, Nik Fariza Husna Nik Hassan, Najib Majdi Yaacob, Norasnieda Md Shukri

**Affiliations:** 1 Department of Otorhinolaryngology-Head and Neck Surgery, School of Medical Sciences, Universiti Sains Malaysia, Kelantan, Malaysia; 2 Department of Otorhinolaryngology, Hospital Sungai Buloh, Selangor, Malaysia; 3 Biostatistics and Research Methodology Unit, School of Medical Sciences, Universiti Sains Malaysia, Kelantan, Malaysia

**Keywords:** smell, olfactory disorder, parosmia, olfaction, questionnaire

## Abstract

**Background:**

Olfactory disorders (OD) are an umbrella term for a diverse group of smell problems. Numerous tests and questionnaires have been formulated to identify and test the severity of smell impairment, which is not readily available or translated for the Malaysian population. This study aimed to translate the Questionnaire for Olfactory Disorders (QOD) and validate and test the reliability of the Malay Questionnaire for Olfactory Disorders (mQOD).

**Methods:**

This cross-sectional study was conducted in two tertiary centres. A forward and backward translation was conducted for the QOD. The translated questionnaire was distributed to subjects with self-reported smell disorders on days 1 and 7. Internal consistency was analysed using Cronbach’s alpha and test-retest reliability was tested with an intraclass correlation coefficient. Confirmatory factor analysis was performed to test construct validity.

**Results:**

A total of 375 participants were recruited, 52 dropped out and 323 completed the questionnaire a second time. The Cronbach’s alpha coefficient was 0.537 for parosmia (P), 0.892 for life quality (LQ), 0.637 for sincerity (S) and 0.865 for visual analogue score (VAS). The intraclass correlation coefficient (ICC) for domain scores was > 0.9, while the ICC for all items was good to excellent. A three-factor model for mQOD showed an acceptable fit with indices chi-square value (CMIN)/degree of freedom (DF) = 3.332, Tucker-Lewis fit index (TLI) = 0.923, comparative fit index (CFI) = 0.939, root mean square error of approximation (RMSEA) = 0.079 and standardised root mean square residual (SRMR) = 0.0574.

**Conclusion:**

The mQOD is a valid and reliable tool for assessing OD in patients.

## Introduction

Olfactory disorders (OD) are becoming increasingly recognised medical problems in the community. Past epidemiological studies have reported that the prevalence of smell disorders ranges from 2.7% to 24.5% (1). The causes of olfactory impairments can be broadly divided into conductive and sensorineural in nature, and at times, they may be a combination of both (2). Conductive causes include chronic sinusitis and nasal polyps, while sensorineural causes include head injury, post-viral upper respiratory tract infection, toxin exposure and age-related decline in smell (1). Recently, coronavirus disease 2019 (COVID-19) has also been identified to cause smell impairments (3–6), triggering renewed interest in OD. The nose plays a major role (7) in causing conductive smell impairment, as it acts as a conduit for odourants and contains neuroepithelium, which is necessary for signal transduction (8). Therefore, it is important for otorhinolaryngologists to anticipate and evaluate this condition in their patients.

The sense of smell is an invaluable sense that enables us to appreciate the scent of objects and, at the same time, acts as a warning signal when encountering potentially hazardous food or environment (9). As a result, smell impairment may reduce quality of life and affect activities of daily living (10). The effects of OD on a person’s life are far reaching, from not being able to smell and enjoy food (10) to reduced self-esteem (11), social isolation (12) and, consequently, depression (13). Patients have reported less enjoyment in food-related activities, such as eating, cooking and going out to eat (14) due to not only loss of smell but also loss of flavour perception. Interestingly, this leads to either increased food intake to compensate for reduced chemosensory perception or reduced food intake as the food becomes less appealing (15). Due to the loss in flavour perception, subjects have been reported to compare their eating experience to eating sawdust and cardboard (12). Patients’ food preferences tend to shift to spicy food to make up for lack of taste and smell (16), along with a change in cooking habits in which they season food more.

Other causes of concern include personal hygiene and social relations (17) as patients worry about body odour or bad breath, which they cannot perceive but may be detected by others. For some patients, a defective chemosensory system can even affect their work (17), especially people in the food industry, perfumers or firemen. Moreover, there is also a close association between olfactory impairment and depression (18). Subjects have smell loss-induced anhedonia and are unable to feel enjoyment (12). A study in Korea showed that subjects with OD were at higher risk of depression and suicidal thoughts (19).

Assessment for olfactory function can be generally categorised into three types: i) psychophysical assessment, ii) imaging studies and iii) subjective assessment (20). Psychophysical tests primarily evaluate odour identification, odour thresholds and odour discrimination. Examples include the University of Pennsylvania Smell Identification Test (UPSIT), the Cross-Cultural Smell Identification Test (CCSIT) and Sniffin’ Sticks. The UPSIT is the most widely known test and has been shown to have high reliability (21). It consists of four booklets with microencapsulated odourants that patients can self-administer. A variant of the UPSIT, CCSIT tests subjects on 12 odours—six food-related and six non-food-related smells. Even though it is a quicker test to administer, it has been shown to be less reliable than the UPSIT. Sniffin’ Sticks uses pen-like odour dispensing devices, which assess all three tests of olfactory function. The downfall of these psychophysical tests is that they cannot be applied universally, as some smells are not known in different parts of the world.

There is a scarcity of questionnaires used to evaluate OD. The Sino-Nasal Outcome Test is a validated questionnaire that explores the burden of disease in chronic rhinosinusitis, but it was not meant to exclusively evaluate OD, as it only has one item pertaining to it. The Questionnaire for Olfactory Disorders (QOD) is a patient-reported questionnaire that aids clinicians in assessing the effect of olfactory impairment on quality of life (22), which has been proven to be a reliable and valid tool in numerous studies. It consists of two parts: i) statements and ii) visual analogue scales (VAS). The statements explore three aspects: i) patient’s life quality (QOD-LQ), ii) sincerity (QOD-S) and iii) parosmia (QOD-P). The VAS makes up the second part of the QOD and gives insights into patients’ awareness of their OD and the degree to which it adversely affects them. To the best of our knowledge, only one exploratory factor analysis (EFA) has been conducted on the QOD. Mattos et al. (23) extracted negative statements (NS) from the QOD, from which the EFA revealed four factors: i) social, ii) anxiety, iii) annoyance and iv) eating-related questions. Confirmatory factor analysis (CFA) can be performed to determine the construct validity of the Malay Questionnaire for Olfactory Disorders (mQOD), which is how well the items measure the construct based on an a priori model. In this case, the constructs were annoyance, anxiety, eating, social and sincerity.

Although loss of smell is not immediately life threatening, it impairs patients’ quality of life in multiple aspects, from eating and cooking habits to interpersonal relationships (14). With the advancement of medical knowledge and technology, we not only try to treat patients’ conditions but also ensure that their quality of life is preserved or improved. By translating the QOD into the Malay language, we hope to provide a validated tool that can be applied to Malaysians. The mQOD can be used as a tool to assess the severity of olfactory dysfunction and monitor symptom improvement.

## Methods

### Research Tool

The latest English version of the QOD was obtained from Johannes Frasnelli. It is made up of 29 statements and 5 VAS. The statements measure three main areas: i) life quality (negative and positive statements, each denoted as QOD-NS and QOD-PS), ii) sincerity and iii) parosmia, with each area consisting of 19, 6 and 4 questions, respectively. The answer for each statement is presented on a Likert scale, with possible answers being ‘I disagree’, ‘I partly disagree’, ‘I partly agree’ and ‘I agree’. The point assigned to each answer is 0, 1, 2 and 3, respectively, except for statements 14, 23, 32, 25 and 36 in which the inverse is true. The maximum raw score for the QOD-LQ is 57, with the formula to convert it into the LQ score being LQ = LQ raw score/0.57 (%). For QOD-S, the maximum raw score is 18, and the formula to convert it into the sincerity score is S = S raw score/0.18 (%). The highest raw score for QOD-P is 12 and the formula for conversion is P = P raw score/0.12 (%). High scores for QOD-LQ and QOD-P indicate strong impairments and parosmia, whereas a low score for QOD-S indicates that the patients may have given what they perceived to be socially acceptable answers. The VAS is a visual scale labelled ‘not at all’ on the left side, denoting a score of 0 and ‘extremely annoying’ on the right side, denoting a score of 10.

### Translation Phase

A forward translation into the Malay language was conducted by two native Malay speakers, a doctor experienced in the management of smell disorders and a professional translator, who were proficient in Malay and English. Backward translation was conducted by another smell disorder expert with no knowledge of the original version of the questionnaire. The back-translation was assessed for equivalence to the original English version by the panel, and a second draft was written. The word ‘accident’ in P1 and P4 was substituted with *gangguan bauan*, which is the Malay equivalent of OD.

Face validity was conducted by testing the second draft on five patients and three medical doctors who were fluent in Malay and English. Any ambiguity or confusion regarding the questions was considered and the feedback form of the mQOD was reviewed. Finally, discrepancies between the original, the forward translation version and the back-translated version were reviewed and reconciled to produce the final mQOD.

### Sample

This was a cross-sectional study in which subjects with self-reported altered smell were recruited through purposive sampling from the otorhinolaryngology clinic and wards of Hospital Universiti Sains Malaysia and Hospital Sungai Buloh. The period of recruitment was from April 2021 to April 2022. Following previous recommendations (24), the target sample size was 300, with a ratio of nine subjects to one item. Considering a dropout rate of 20%, the final sample size was inflated to 375 participants. The inclusion criteria included individuals with impaired smell aged 18 years old and above and fluent in the Malay language. Patients with cognitive disturbances and speech and hearing difficulties were excluded. The subjects were informed about the need to complete the questionnaire a second time. The patient information sheets and questionnaires were made available as printouts and Google Form links. On day 1, the questionnaires were answered on physical forms in front of the researchers. The mQOD was administered again to the same participants after a 7-day interval via Google Form links, which were sent to their mobile phones.

### Data Analysis

Data analysis was performed using the Statistical Package for the Social Sciences version 28.0 and CFA was conducted using analysis of moment structures. Data from day 1 were analysed for internal consistency and construct validity, while test-retest reliability was analysed using data from days 1 and 7. Internal consistency was measured using Cronbach’s alpha, with acceptable values of 0.5–0.7 and good values of 0.7–0.8 (25). Test–retest reliability was determined using intraclass correlation coefficient (ICC), where ≤ 0.5 means poor, 0.5–0.75 means moderate, 0.75–0.9 means good and ≥ 0.90 means excellent reliability (26). Test-retest reliability analysis was conducted on 157 respondents without viral or post-viral infections to ensure temporal consistency.

We created a five-factor model by adding a sincerity factor to the four-factor model, as suggested by a previous study (23). The remaining items of the questionnaire were distributed within a pre-specified model. The factors and their items are as follow:

- Social-related questions (SOC): q20, q21, q25, q26, q28, q29, VAS3 and VAS4
- Eating-related questions (EAT): q1, q5, q7, q17 and q24
- Annoyance-related questions (ANN): q2, q3, q4, q6, q8, q13, q22, VAS1, VAS2 and VAS5
- Anxiety-related questions (ANX): q10, q12, q15, q16 and q16
- Sincerity questions (SIN): q9, q11, q14, q18, q23 and q27.

The acceptable factor loading was set to ≥ 0.4 (27). The following are the parameters of good fit: CMIN/DF ≤ 5 comparative fit index (CFI) and Tucker-Lewis fit index (TLI) > 0.9, root mean square error of approximation (RMSEA) < 0.08 and standardised root mean square residual (SRMR) < 0.08 (28). Convergent validity was measured by the average variance extracted (AVE), which should be > 0.50 (29). Composite reliability (CR) is a measure of construct reliability, which was set to > 0.7 (30). The AVE and CR values were calculated following Fornell and Larcker’s equations (31).

## Results

### Demographics

A total of 375 subjects were recruited for this study; among these, 52 dropped out and did not complete the questionnaire on day 7. Our sample comprised 181 (48.3%) men and 194 (51.7%) women. The youngest subject was 18 years old and the oldest was 69 years old, with a mean age of 35.9 years old. Almost half of the subjects had post-viral infection, including COVID-19 (47.5%) and asthma, allergic rhinitis or eczema (27.7%). Table 1 summarises the subject demographics.

### Reliability

The Cronbach’s alpha coefficient was 0.537 for P, 0.891 for LQ, 0.637 for S and 0.865 for VAS. The overall internal consistency was good at 0.897. The ICC was 0.975, 0.979, 0.979 and 0.979 for P, LQ, S and VAS scores, respectively. All items of each domain had an ICC > 0.9, except for questions 5 and 17 (> 0.8), with a *P*-value < 0.001. Tables 2 and 3 summarise the tests of reliability for mQOD.

### Validity

Our data showed a poor fit with the five-factor model, and there were high factor correlations between ANN and EAT (0.801) and between SOC and ANX (0.855). Therefore, these factors were combined and the model was converted into a three-factor model. The following items with factor loadings < 0.4 were deleted:

- q27: Sometimes I talk about things I do not understand.
- q2: Sometimes I think I can smell something bad, even when other people cannot.
- q3: Some of the smells that I find unpleasant, other people find pleasant.
- q19: I can imagine adjusting to the changes in my sense of smell.
- q5: Because of the changes in my smell, I go to restaurants less often than I used to.
- q4: One of my biggest problems is that smells are different from what they used to be before my accident.
- q17: Because of the changes in my sense of smell, I have weight problems.
- q11: Sometimes I have thoughts and ideas that I do not want other people to know.
- q15: Because of the changes in my sense of smell, I visit friends, relatives or neighbours less often.

The following three covariances were made based on the modification indices:

- VAS1 (Rate how annoying the changes in smell are) and VAS2 (Rate how often you become aware of the changes to your sense of smell)
- VAS2 (Rate how often you become aware of the changes in your sense of smell) and q6 (I am always aware of the changes in my sense of smell)
- q7 (Because of the changes in my sense of smell, I do not enjoy drinks or food as much as I used to) and q13 (The changes in my sense of smell annoy me when I am eating)

**Figure 1:**
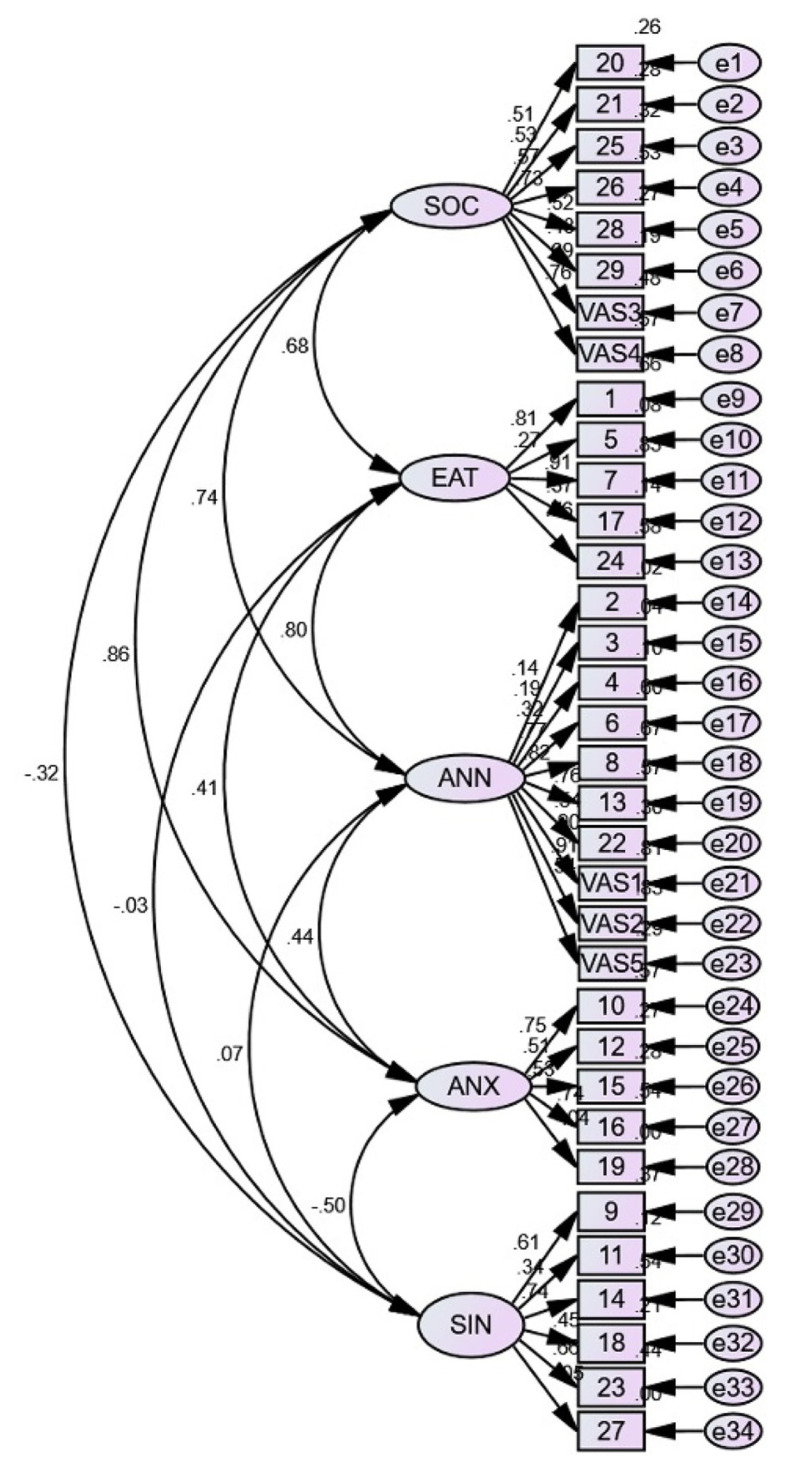
Five-factor model for mQOD Notes: SOC = social related questions, EAT = eating related questions, ANN = annoyance related questions, ANX= anxiety related questions, SIN = sincerity related questions

**Figure 2:**
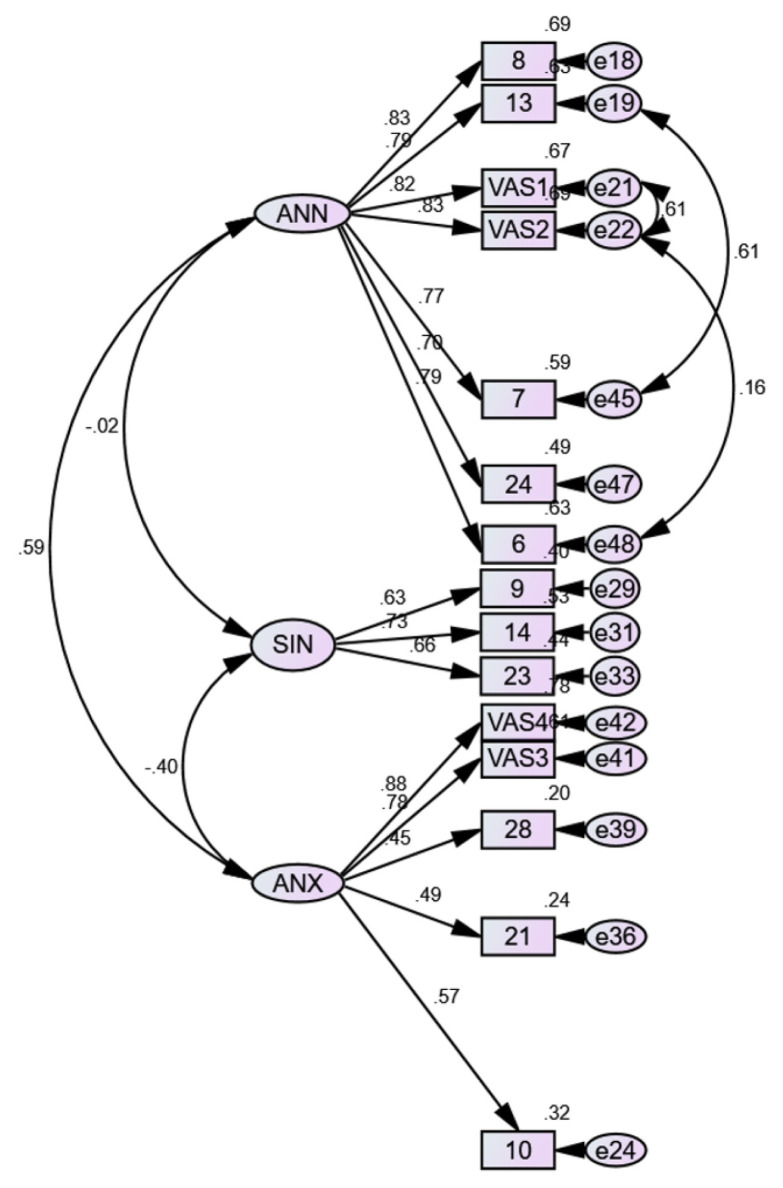
Three-factor model for mQOD Notes: SOC = social related questions, EAT = eating related questions, ANN = annoyance related questions, ANX= anxiety related questions, SIN = sincerity related questions

Nine items with standardised residual covariance ≥ 2 were removed: q1, q25, VAS5, q26, q29, q16, q20, q12 and q18. The final three-factor model with 15 items had an acceptable model fit with CMIN/DF = 3.332, TLI = 0.923, CFI = 0.939, RMSEA = 0.079 and SRMR = 0.0574. Table 4 shows the fit statistics for the five-and three-factor models, and Figures 1 and 2 show the diagrams of the models.

The remaining items had factor loadings of 0.490–0.883. The CR for all factors was > 0.7, and the AVE was 0.627 for ANN, 0.454 for SIN and 0.431 for ANX. The correlation was 0.02 between ANN and SIN, −0.40 between SIN and ANX and, 0.59 between ANX and ANN (Table 5).

## Discussion

Since its conception in 2005, the QOD has been translated into multiple languages and has proven to be valid and reliable in numerous studies. In this study, we aimed to test the validity and reliability of the mQOD so that it could be applied to the majority Malay-speaking population in Malaysia. The mQOD can also be a useful tool in the clinical setting to assess the burden of disease in patients and to monitor the treatment response.

In this study, gender distribution was almost equal, but women had higher mean scores in the LQ, P and VAS domains. This finding is consistent with other studies (32, 33), suggesting that women are more affected by smell impairment. The mean scores for all domains decreased on day 7, indicating improvement in symptoms. Our results showed that the LQ statements and VAS components had good internal consistency, with Cronbach’s alphas of 0.891 and 0.865. Cronbach’s alphas for the P and S statements were 0.545 and 0.637, respectively, which is considered acceptable. The Turkish, Korean and Persian translations of the QOD also showed a lower Cronbach’s alpha for the S statements, with 0.62, 0.243 and 0.25, respectively (34–36). The S statements were initially designed as a measure of the subject’s credibility (22) and although this scale may be relevant in psychological studies, these statements may be omitted in a clinical setting for the sake of time efficiency, as their removal does not alter much the overall internal consistency. The Chinese equivalent of the QOD also showed a low internal consistency for the P statements, with 0.473, citing cultural differences (37). Another reason for this could be the low number of items or the poor interrelation between the items (38). This poor interrelation is supported by the fact that when the P1 statement (Food tastes different than it used to be) was removed, it increased the Cronbach’s alpha of the P domain to 0.757.

In test-retest studies, the time frame needs to be long enough so that participants will not recall the test items but short enough so that there will not be changes in their condition (39). Other studies have reported intervals of 2 weeks to 12 months between the test periods (33, 34, 37, 40, 41). In our study, the mQOD was readministered after a 7-day interval. The ICC for the domain scores and their items were all excellent, indicating that the mQOD is a reliable measure. Our findings echo Lechien et al. (42)’s study, which had excellent test–retest reliability, except for the S domain. The anglicised version of the QOD showed moderate to good agreement measures of 0.68–0.78 (33). Similarly, Yang et al. (37) showed good correlations in all domain scores except for VAS.

Our data fit poorly in the initial five-factor model. The assessment of the model fit revealed several factors that were highly correlated and later combined. The factors SOC and ANX were combined because they both measure anxiety, while annoyance was the common latent variable underlying ANN and EAT. Error covariances were made based on modification indices to improve the model fit. VAS1 and VAS2 were worded similarly, while q6 and VAS2 both measured the frequency with which the subjects realised their olfactory impairment. Lastly, q17 and q13 both explored the effect of OD on their eating experience. Although arguments have been made against post hoc error term correlations (43, 44), some still consider it legitimate in similarly worded test items (45). Although the AVE value for SIN and ANX was < 0.5, their CR was > 0.7, which demonstrates acceptable convergent validity (46). The factor correlations were < 0.85, which shows good discriminant validity (28). This means that the latent variables are unrelated and measure distinct constructs.

This study has several limitations. The subjects were recruited based on self-reported olfactory impairment and no objective smell evaluation was conducted. Therefore, we were unable to verify whether all the subjects had OD. Furthermore, due to the COVID-19 pandemic, there was a disproportionate number of subjects with post-viral infection, which could implicate the generalisability of the study findings. In the future, another validation study should be performed on a new sample set, as more than 20% of the original items have been deleted (47).

## Conclusion

The mQOD is a valid and reliable tool for measuring smell impairment.

## Figures and Tables

**Table 1:** Subject demography

Variables	*n*	%	Mean ± SD
Gender			
Male	181	48.3	
Female	194	51.7	
Age			35.97 ± 9.41
Race			
Malay	309	82.4	
Chinese	38	10.1	
Indian	15	4.0	
Others	13	3.5	
Duration of symptoms			
< 1 month	121	32.3	
> 1 month	120	32.0	
> 6 months	32	8.5	
> 1 year	102	27.2	
Comorbidities			
Allergies			
Yes	40	10.7	
No	335	89.3	
Smoker			
Yes	36	9.6	
No	339	90.4	
Tumour			
Yes	52	13.9	
No	323	86.1	
Asthma/Allergic Rhinitis/Eczema			
Yes	104	27.7	
No	271	72.3	
History of Rhino surgery			
Yes	26	6.9	
No	349	93.1	
Trauma/Brain surgery			
Yes	13	3.5	
No	362	96.5	
Post-viral infection (COVID-19)			
Yes	178	47.5	
No	197	52.5	

**Table 2:** Cronbach’s alpha coefficient

	P	LQ	S	VAS	Overall
Cronbach’s alpha	0.545	0.891	0.637	0.865	0.897

**Table 3:** Test retest reliability

Items	ICC
P score	0.975
1	0.972
2	0.979
3	0.943
4	0.928
LQ score	0.979
5	0.880
6	0.937
7	0.975
8	0.938
10	0.961
12	0.946
13	0.962
15	0.945
16	0.928
17	0.875
19	0.953
20	0.941
21	0.939
22	0.962
24	0.979
25	0.969
26	0.976
28	0.939
29	0.912
S score	0.979
9	0.989
11	0.985
14	0.966
18	0.949
23	0.979
27	0.940
VAS score	0.979
VAS 1	0.967
VAS 2	0.945
VAS 3	0.882
VAS 4	0.902
VAS 5	0.977

**Table 4:** Goodness-of-fit statistics for the models

Model	CMIN/DF	TLI	CFI	RMSEA	SRMR
Five-factor	9.011	0.553	0.593	0.146	0.1528
Three-factor	3.332	0.923	0.939	0.079	0.0574

**Table 5:** Factor loadings, composite reliability and average variance extracted of three-factor model

Construct	Item	Factor loading	Composite reliability	Average variance extracted
ANN	q6	0.794	0.922	0.627
	q7	0.770		
	q8	0.833		
	q13	0.793		
	q24	0.700		
	VAS1	0.818		
	VAS2	0.830		
SIN	q9	0.630	0.714	0.454
	q14	0.727		
	q23	0.662		
ANX	q10	0.567	0.780	0.431
	q21	0.490		
	q28	0.453		
	VAS3	0.782		
	VAS4	0.883		
